# Epstein-Barr Virus in Systemic Autoimmune Diseases

**DOI:** 10.1155/2013/535738

**Published:** 2013-08-24

**Authors:** Anette Holck Draborg, Karen Duus, Gunnar Houen

**Affiliations:** Department of Clinical Biochemistry, Immunology and Genetics, Statens Serum Institut, Artillerivej 5, 2300 Copenhagen, Denmark

## Abstract

Systemic autoimmune diseases (SADs) are a group of connective tissue diseases with diverse, yet overlapping, symptoms and autoantibody development. The etiology behind SADs is not fully elucidated, but a number of genetic and environmental factors are known to influence the incidence of SADs. Recent findings link dysregulation of Epstein-Barr virus (EBV) with SAD development. EBV causes a persistent infection with a tight latency programme in memory B-cells, which enables evasion of the immune defence. A number of immune escape mechanisms and immune-modulating proteins have been described for EBV. These immune modulating functions make EBV a good candidate for initiation of autoimmune diseases and exacerbation of disease progression. This review focuses on systemic lupus erythematosus (SLE), rheumatoid arthritis (RA), and Sjögren's syndrome (SS) and sum up the existing data linking EBV with these diseases including elevated titres of EBV antibodies, reduced T-cell defence against EBV, and elevated EBV viral load. Together, these data suggest that uncontrolled EBV infection can develop diverse autoreactivities in genetic susceptible individuals with different manifestations depending on the genetic background and the site of reactivation.

## 1. Systemic Autoimmune Diseases

Systemic autoimmune diseases (SADs), also called rheumatic connective tissue diseases, include rheumatoid arthritis (RA), Sjögren's syndrome (SS), systemic lupus erythematosus (SLE), mixed connective tissue disease (MCTD), systemic scleroderma (SSc), and dermatomyositis/polymyositis (DM/PM). SADs are characterized by overlapping clinical symptoms and characteristic autoantibodies (Table 1). Some of the most extensively studied SADs are SLE, RA, and SS, and this review will focus on these.

The etiology of SADs is believed to be multifactorial with both genetic and environmental factors contributing to the disease development. Concordance has been observed in monozygotic twins, and specific genes including some coding for certain major histocompatibility complex (MHC) II alleles have been shown to be associated with development of these diseases [1–5]. The major environmental risk factors for SAD development are infections, including Epstein-Barr virus (EBV) infection which is suspected to have a central role in the pathogenesis of SADs as presented in the later sections of this review. Furthermore, EBV has for decades been associated with induction of various cancers, including lymphoid malignancies (e.g., Burkitt's lymphoma [6]) and epithelial cell malignancies (e.g., nasopharyngeal carcinoma [7]). 

### 1.1. SLE

SLE is a rare autoimmune disease with a prevalence of 0.09% and an incidence of 1–10 new cases per 100.000 per year, and nine out of 10 patients are women [8–12]. Typical symptoms involve the butterfly rash at the malar region of the face, photosensitivity, oral- and nasopharyngeal ulcers, arthritis, renal and hematologic disorders, and autoantibodies against nuclear components. The clinical presentation of SLE is influenced by a variety of factors including ethnicity, gender, age, and age of onset [8]. The typical course of the disease is demonstrated by periods of disease flares alternating with remission.

Various immune-deficiencies have been identified in SLE patients. Abnormalities in the complement cascades are observed in some SLE patients. C1q and C4 deficiencies serve as severe risk factors for development of SLE with a risk of developing SLE at 93% and 75%, respectively. C1q deficiency can also be acquired as a result of production of C1q autoantibodies, which are detected in 40–50% of SLE patients. Deficiencies in the complement system result in decreased clearance of apoptotic material, which may initiate autoimmune responses and production of autoantibodies against cellular components [13–16]. Additionally, SLE is characterized as an immune complex disease comprising autoantibodies and their specific autoantigens. These will deposit in the subendothelium, when the concentration and size reach a critical level and cause inflammation and tissue damage [15, 17].

### 1.2. RA

RA is a common autoimmune disease with a prevalence of approximately 1% and an incidence of 5–50 per 100,000 per year with three times more female than male patients [18]. Common symptoms include arthritis, cardiovascular complications, metabolic syndrome, cognitive dysfunction, and depression. Furthermore, involvement of the lungs, kidneys, and skin are observed in RA patients [18, 19].

Another environmental risk factor for development of RA, besides infections, is smoking and other forms of pulmonary stress. Environmental stress may promote posttranslational modifications of proteins including citrullination via peptidylarginine deiminases [19]. Loss of tolerance will thereby result in autoantibodies against these citrullinated proteins (CCP antibodies) characteristic of RA patients (Table 1).

Several inflammatory processes are involved in the disease course. Most of all, the persistent synovial inflammation with infiltration of macrophages, T- and B-cells, immune complexes, and a variety of cytokines result in joint damage and cartilage destruction, ultimately leading to impaired movement and deformity of involved joints [18]. Furthermore, prolonged inflammation also leads to bone erosion by promoting osteoclast differentiation resulting in osteoporosis and bone fractures [19]. 

### 1.3. SS

SS is a rather common autoimmune disease with a prevalence of about 0.5% and an incidence of 3–6 per 100,000 per year with a female preponderance (nine out of 10 SS patients are women) [26, 27]. It may present as primary SS, but it can also be associated with other SADs including SLE and RA. 

SS is characterized by disorders of exocrine glands (particularly salivary and lacrimal glands resulting in dry eyes and dryness of mouth) with presence of infiltrating lymphocytes, dysfunction of muscarinic receptors, chronic inflammation, and development of specific autoantibodies (Table 1). Furthermore, several extraglandular manifestations are observed in SS patients, including dry skin, pancreatitis, gastritis, arthritis, neurosensory deafness, serositis, pulmonary fibrosis, hypergammaglobulinemia, and involvement of kidneys and the nervous system [27].

Presumably, the initial pathogenic steps in the development of SS involve changes in the glandular epithelial cells, including cell death giving rise to upregulation of adhesion molecules and chemokines, which stimulate lymphocyte migration to the glands resulting in lymphocyte extravasation, infiltration, and glandular destruction [26]. Actually, E-cadherin, an epithelial cell adhesion molecule, has been demonstrated to be increased in patients with SS suggesting enhanced adhesion of lymphocytes to epithelial cells tissue [28]. The systemic manifestations in SS presumably occur upon lymphocyte infiltrations in other tissues and also as a result of pathogenic autoantibodies.

## 2. EBV

EBV is a ubiquitous infectious agent, latently infecting approximately 95% of the world's population [29]. Primary infection with EBV mostly occurs during childhood and causes a mild, usually asymptomatic infection. However, primary infection in adolescence causes infectious mononucleosis (IM) in 30–70% of cases, where up to 20% of B-cells are infected with EBV [30, 31]. This age-related difference in disease progression has yet to be explained [32]. 

**Figure 1:**
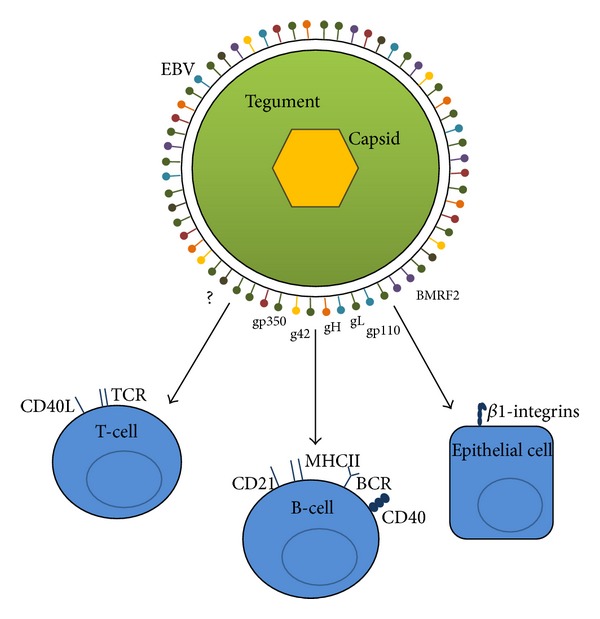
Epstein-Barr virus structure and infection of cells. Epstein-Barr virus (EBV) is comprised of a dsDNA genome inside an icosahedral capsid which is surrounded by the tegument and enclosed by a host cell membrane-derived envelope. During infection with EBV, different envelope glycoproteins (gps) (shown in different colors) induce viral entry. During viral entry of B-cells, viral gp350 binds to type 2 complement receptor (CD21) on B-cells, and via a complex of gp42, gH, and gL, fusion of the cell membrane and the viral envelope is induced through (major histocompatibility complex) MHC II on the B-cell. During viral entry of epithelial cells, viral BMRF2 binds to *β*1-integrins on the epithelial cell, and fusion of the membranes is facilitated by a complex of gH and gL. gp110 improves the efficiency of EBV to infect B-cells and epithelial cells. EBV can also infect T-cells; however, the mechanism of viral entry is unknown (?). BCR: B-cell receptor, TCR: T-cell receptor.

EBV is a DNA virus of the herpes family (human herpesvirus 4). It is comprised of a linear dsDNA genome enclosed by an icosahedral capsid, which is surrounded by the tegument and a host cell membrane-derived envelope embedded with glycoproteins (gps) (Figure 1). EBV has a fairly large genome coding for 87 proteins, and the functions of 72 of these are so far elucidated [33].

EBV is transmitted in saliva and initially infects epithelial cells in the oropharynx and nasopharynx. Subsequently, EBV enters the underlying tissues and infects B-cells [34, 35]. After primary lytic infection, EBV persists in immortalized resting memory B-cells for the rest of the individual's life and can shift between an active lytic cycle and a latent state, from which it occasionally reactivates [36]. This ability of the virus to reactivate makes it a constant challenge to the host.

In the latent state, the EBV genomic DNA will undergo circularization and replicate together with the host's chromosomal DNA, which results in a restricted expression of viral genes and conceals the virus from the host's immune system [34, 35]. During the latent state, a maximum of nine genes are expressed including the EBV nuclear antigens (EBNA1, -2, -3A, -3B, and -3C), the leader protein (LP), and the latent membrane proteins (LMP1, -2A, and -2B) [36, 37] (Table 2). EBNA1 is the only protein required for maintenance of the viral genome serving as a replication factor. When B-cells are latently infected for longer periods of time, EBV will only express EBNA1 [34–36]. EBNA2 is an important transcription factor during latency as it controls the expression of all other latent viral genes [36]. LMP1 and LMP2A rescue the infected B-cells from apoptosis, as they deliver the signal that normally comes from the CD40 signal transduction pathway initiated by CD4+ T-cell help and provide the signal normally generated by antigen binding of the B-cell receptor, respectively [37, 38].

The exact triggers for lytic cycle reactivation are unknown, but the process is a dynamic interaction between the host's immune response to EBV and the infection state. Activation of the promoter for the early lytic genes and, thereby, initiation of lytic replication are triggered by the differentiation of infected B-cells into plasma cells [34, 36, 40, 41]. 

During lytic cycle of infection, EBV expresses numerous proteins involved in different viral activities. In the induction of lytic replication, two transcription factors, BZLF1 and BRLF1, activate early viral promoters required for generation of the initiation complex consisting of six viral proteins (Table 2): the viral DNA polymerase (BALF5), the viral DNA polymerase accessory protein, early antigen diffuse (EA/D), a single-stranded DNA-binding protein (BALF2), the primase (BSLF1), the helicase (BBLF4) and the helicase/primase-associated protein (BBLF2/3) [42–47]. The binding of BZLF1 and the gathering of the initiation complex at the lytic origin of replication, *oriLyt*, result in multiple viral genome replication cycles with a 100- to 1000-fold amplification [35, 46] and expression of lytic genes [33]. After synthesis of viral DNA, various viral proteins induce packaging and encapsidation of the viral genome, which is subsequently released from the nucleus to the cytoplasm of the infected cell. In the tegument, various viral enzymes induce assembly, envelopment, and glycosylation of the virion. Ultimately, new infectious virions are produced and shed from the cell. These can infect other cells and can also be transmitted to a new host [36].

New virions primarily infect B-cells and epithelial cells, but various other cell types, including T-cells and natural killer cells, can also be infected [48–50]. During viral entry of B-cells, viral gp350 binds to the B-cell type 2 complement receptor (CD21) [34, 35]. A complex of three gps (gp42, gH, and gL) induces fusion of the viral envelope with the cell membrane by binding to MHC II. Viral entry of epithelial cells is induced via binding of viral BMRF2 to *β*1 integrins and similarly to fusion with the B-cell membrane, a complex of gH and gL facilitates fusion of the viral envelope. Furthermore, gp110 improves the efficiency of the virus to infect both B-cells and epithelial cells [51] (Figure 1) (Table 2). The mechanism of viral entry in T-cells is unknown. However, it could be speculated that some of the envelope proteins (Table 2) with unknown function may be implicated in viral entry in T-cells and possibly other immune cells.

Several EBV proteins are involved in immune evasion (Table 2) mainly by inhibiting the interferon (IFN) pathways and T-cell immunity. An example is the viral interleukin(IL)10 homologue, which, like human IL10, inhibits the synthesis of IFN*γ* and suppresses CD8+ cytotoxic T-cell responses and the upregulation of MHC I expression [52]. Furthermore, viral antiapoptotic proteins are expressed during lytic cycle of infection including early antigen restricted (EA/R), which is a viral Bcl2 homologue that protects both infected B-cells and epithelial cells from apoptosis [53].

## 3. EBV in SADs

### 3.1. EBV in SLE

Many studies have linked EBV to the development of SLE. SLE patients have been shown to have an abnormally high viral load in the peripheral blood mononuclear cells (PBMCs) compared to healthy controls with 10–40-fold increase [54–58]. The viral load was found to be associated with disease activity and to be independent of intake of immunosuppressive medication. Furthermore, an elevated level of EBV DNA was found in serum from 42% of SLE patients compared to only 3% of healthy controls [56]. The findings on increased EBV load suggest active EBV lytic replication in SLE patients. As the viral load was associated with disease activity, it could be speculated that the reactivation of EBV is associated with development of SLE and flares.

Usually, little or no mRNA expression by EBV is observed in normal immune competent carriers of EBV. However, several groups have demonstrated that SLE patients have abnormally high expression of several viral mRNAs (coding for BZLF1, gp350, viral IL10, LMP1, LMP2, and EBNA1) [54, 59]. High expression of BZLF1 could imply reactivation of EBV, and increased gp350 could be speculated to result in an amplified number of B-cells being infected with EBV. Furthermore, increased expression of viral IL10 may give rise to enhanced immune evasion from the cell-mediated part of the immune system. In addition, an abnormal EBV latent state is also indicated by these results with improved survival of infected cells via enhanced expression of the LMP's [54, 59].

Much serologic evidence of a connection between EBV infection and SLE has been demonstrated. Antibodies to EBNA1, viral capsid antigen (VCA), and EA in sera from SLE patients have been examined. Most studies find no difference between SLE patients and healthy controls in the prevalence of IgG and IgM antibodies to either EBNA1 and VCA [60–63], but studies on pediatric SLE patients and one study on adults show that all SLE patients are seropositive for these antibodies compared to two-thirds of healthy controls [29, 64, 65]. Furthermore, elevated titers of IgG antibodies to EA/D, EA/R, and BALF2 have been observed in about half of SLE patients compared to only 8–17% of healthy controls [60, 62, 63, 66, 67]. Additionally, high levels of IgA antibodies to EA/D have been found in 58% of SLE patients and not in healthy controls [68, 69]. These results could not be explained by immunosuppressive medication, indicating that the antibodies are not produced upon reactivation of EBV due to an iatrogenically suppressed immune system. Presumably, these results reflect the host's attempt to control reactivation or reinfection of EBV in epithelial cells [68].

EBV infection is mainly controlled by cell-mediated immunity. However, EBV-specific cytotoxic T-cell reactivity has been observed to be reduced in SLE patients resulting in poor control of the EBV infection. Less CD8+ cytotoxic T-cells were found to produce IFN*γ* upon stimulation with EBV in the SLE patients compared to healthy controls, which must be a consequence of either defective or fewer EBV-specific cytotoxic T-cells [55, 70, 71]. 

Thus, SLE patients have an elevated viral load, increased EBV mRNA expression, elevated levels of EBV-directed antibodies, and decreased EBV-directed cell-mediated immunity compared to healthy controls, indicating poor control of EBV with frequent reactivation.

### 3.2. EBV in RA

EBV has for long been suspected to have a role in the pathogenesis of RA. By the use of several methods including in situ hybridization and PCR, presence of EBV DNA/RNA has been demonstrated in PBMCs, saliva, synovial fluid, and synovial membranes of RA patients [72–76]. Furthermore, 10-fold higher frequencies of EBV-infected B-cells have been observed in RA patients compared to healthy controls [77]. Interestingly, EBV DNA was found in many of the plasma cells producing CCP antibodies localized in synovial tissues of RA patients [78]. These results indicate a widespread lytic EBV infection in RA patients, that also localize in the joints, suggesting a role for EBV-infected cells in the synovial inflammation characteristic for RA patients [78].

In addition, studies on EBV antibodies have shown a humoral response to both latent and lytic EBV antigens with elevated titers of antibodies against EBNA1, VCA, and EA/R in both sera and synovial fluids from RA patients compared to healthy controls [72, 79–81]. 

Investigations on EBV-specific T-cells in the peripheral blood of RA patients have revealed a defective IFN*γ* response to EBV proteins compared to healthy controls [82]. A study regarding gp110-specific T-cells in the peripheral blood showed that T-cells from RA patients had a decreased response to gp110 compared to healthy controls, and this was associated with disease activity [83]. As gp110 is important in viral entry during infection of B-cells and epithelial cells, a decreased gp110-specific T-cell response could be speculated to reduce the control of EBV and also enhance spreading of the EBV infection in RA patients. Contrarily, CD8+ cytotoxic T-cells specific for the two lytic cycle EBV antigens, BZLF1 and BMLF1, have been detected in synovial fluid and synovial membranes of RA patients, indicating a contribution of infiltrated cytotoxic T-cells specific for EBV lytic cycle antigens in joint inflammation [84, 85].

Thus, research has revealed increased viral load, high titers of EBV-directed antibodies, and decreased cell-mediated control of EBV in RA patients compared to healthy controls and suggested a role for infiltrated EBV-specific T-cells in synovial inflammation of RA patients. 

### 3.3. EBV in SS

EBV infection has also been associated with SS, with findings of increased viral load [86–90] and EBV-directed antibodies in SS patients [91–94]. Furthermore, SS patients are known to have an increased risk of development of EBV-associated lymphomas, additionally indicating this association [95]. About 5% of SS patients will develop a lymphoid malignancy, in most cases (mucosa-associated lymphoid tissue) MALT lymphoma in the salivary gland or non-Hodgkin's lymphoma [95]. 

One study has shown that saliva from SS patients is able to activate EBV. Eight out of 12 SS saliva samples were found to have an activating effect on the *BZLF1* promoter in EBV-negative *BZLF1-*transfected salivary gland cells, indicating a possible frequent reactivation of EBV in the oropharynx of SS patients [96].

High loads of EBV DNA have been observed in saliva from SS patients and in both infiltrated B-cells and epithelial cells in salivary glands from SS patients [86, 87, 89, 90]. Furthermore, by the use of a monoclonal antibody directed against the lytic cycle antigen EA/D, a cytoplasmic staining of epithelial cells in salivary glands has been observed in 57% (eight of 14) of SS patients compared to none of the healthy controls [86]. These results suggest EBV reactivation in the epithelial cells in salivary glands of SS patients, which could initiate an immune response that damages the salivary glands of SS patients.

Moreover, EBV DNA has been observed in the lacrimal glands of SS patients and EBV latent and lytic proteins were detected by the use of immunohistochemistry in areas with B-cells and epithelial cells in lacrimal gland tissue from SS patients and not in the healthy controls [88]. Thus, EBV may also play a role in the lacrimal gland disorders characteristic of SS patients.

Studies have shown elevated levels of antibodies against EBNA, VCA, and EA in serum from SS patients [91, 92, 94]. One study demonstrated IgG antibodies directed against EA/D in 36% (36 of 100) of SS patients compared to only 4.5% of healthy controls. The presence of these antibodies was not associated with intake of immunosuppressant medication [93]. 

Thus, increased viral load and EBV proteins have been found in salivary and lacrimal glands of SS patients indicating active infection, and elevated levels of EBV-directed antibodies have been found in the circulation. 

## 4. Genetic Factors and Possible Mechanisms Associated with Induction of Autoimmunity

Much investigation has suggested an etiologic role for active and uncontrolled EBV infection in development of the SADs in genetically predisposed individuals. This is demonstrated by defective EBV-specific T-cells, increased viral loads and elevated expression of lytic cycle proteins, and high levels of antibodies against EBV in SLE, RA, and SS patients [29, 54–58, 60–68, 70–83, 86–94]. These findings suggest widespread infection and frequent EBV reactivation in SLE, RA, and SS patients.

EBV is a good candidate for a causal agent in SADs. EBV has the ability to persist in the host as a latent infection that occasionally reactivates, which presumably contributes to the disease flares observed in the chronic SADs. EBV-induced IM has similar symptoms and clinical manifestations as the individual SADs, including presence of rheumatoid factor and other autoantibodies [32, 97–99], and primary acute EBV infection is also known to induce production of nuclear autoantibodies characteristic of SADs [100]. Furthermore, inoculated EBV infection in humanized mice has been demonstrated to generate RA-resembling arthritis [101].

Several mechanisms have been associated with the induction of autoimmunity by EBV [102]. EBV infection influences the host's immune system both directly through infection of various lymphocytes (for instance will the infection of B-cells possibly result in proliferation, enhanced antibody production, and formation of immune complexes [102]) and indirectly by expression of numerous immune-modulating proteins [36–38, 52, 53, 102]. EBV proteins involved in immune evasion and suppression of apoptosis of transformed infected lymphocytes are likely to result in loss of tolerance and development of autoimmunity [37, 38, 52, 53].

EBV is able to stimulate the innate immune system via EBV-encoded small RNA (EBER) in complex with La (SSB) through Toll-like receptor 3 and thereby induce production of inflammatory cytokines [103]. Thus, in this way, EBV might enhance the autoreactivity against the ribonucleoprotein La (SSB) often found in SS and SLE. Furthermore, bystander activation and expansion of autoreactive T-cells are known to occur due to the virus-induced severe local inflammation and intense local cytokine production [102].

An additional mechanism by which EBV may contribute to loss of tolerance and development of autoimmunity is molecular mimicry [102]. EBNA1 has been shown to cross-react with the autoantigen Ro (SSA) resulting in cross-reactive antibodies followed by epitope spreading [59, 65, 93, 104, 105].

EBV could be involved in SLE, RA, and SS through both common and different genetic or acquired immune-deficiencies connecting EBV to these overlapping yet different diseases. Common gene variants known to be involved in the pathogenesis of all three diseases include components of cytokine pathways (e.g., *IRF5*,* STAT4,* and *TNFSF4*) [2, 3, 19, 106–108] presumably contributing to the development of systemic autoimmunity. In addition, several individual gene variants are associated with the pathogenesis of the specific diseases. Especially gene variants involved in the complement system (including *ITGAM*) are specific for SLE patients [3, 109], and gene variants involving the muscarinic receptors (*CHRM3*) are specific for SS patients [110]. Gene variants specific for RA patients include *PADI4* variants coding for the enzyme that catalyzes the citrullination of arginine residues of proteins [19]. Thus, genetic (and epigenetic) variations may contribute to specific immune-deficiencies and, thereby, altered immune response to EBV and altered control of EBV infection [2, 3, 19, 106–110]. The constant interplay between EBV reactivation and the host's immune response probably results in individual disease patterns and clinical manifestations according to the genetic background, site of reactivation or reinfection and type of infected cell [3, 19, 36, 40, 48, 110].

In SS patients, studies show that the EBV replication is mainly localized in epithelial cells and infiltrated B-cells of the salivary and lacrimal glands [86–90, 96]. In RA patients, the EBV-infected cells in the synovial joints are shown to undergo frequent reactivation including EBV-infected plasma cells producing CCP autoantibodies [72, 74–76, 78, 84, 85]. The EBV reactivation results in production of lytic cycle proteins giving rise to host immune responses that presumably contribute to the inflammation and destruction of exocrine glands and synovial joints in SS and RA patients, respectively [72, 74–76, 78, 84–90, 96]. In SLE patients, EBV reactivation of epithelial cells could be involved in the symptoms of skin and mucosa [68]. Furthermore, a systemic EBV reactivation of both B-cells and epithelial (and possibly other cell types) may occur, giving rise to the various overlapping systemic manifestations observed in the three SADs. Reactivation of EBV and thereby an increased number of EBV-infected cells presumably result in increased amounts of cellular waste and thus stimulation of autoreactive B-cells and consequently production of autoantibodies resulting in disease flares. For each reactivation, the amounts of EBV immune evasion and antiapoptotic molecules expand, resulting in a vicious circle of increased disease activity [35, 46].

## 5. Conclusion

In conclusion, EBV is demonstrated to have a role as an environmental trigger in the development of SADs. Individual genetically determined and acquired differences in innate and adaptive immunity and the constant interplay between the host's immune response and EBV immune-modulating proteins may result in individual disease patterns, which are overlapping, but clinically may be classified as SLE, RA, and SS. It is also possible that every time the control of EBV diminishes, EBV reactivates and reinfects more cells of different types in different locations of the body, resulting in specific manifestations and progression of disease flares [3, 19, 36, 38, 48, 52, 53, 110].

## Figures and Tables

**Table 1:** Prevalence (%) of autoantibodies in RA, SS, and SLE.

	CCP	RF	Ro52	Ro60 (SSA)	La (SSB)	dsDNA	ANA	References
RA	50–80	70–80	5–10	5–10	0–5	0–10	30–50	[20–22]
SS	5–10	40–70	20–40	40–80	30–60	0–10	40–70	[21, 23, 24]
SLE	5–10	20–30	10–20	20–40	15–20	70–80	95–100	[9, 21, 25]

RA: rheumatoid arthritis; SS: Sjögren's syndrome; SLE: systemic lupus erythematosus; CCP: cyclic citrullinated peptide; RF: rheumatoid factor; dsDNA: double-stranded DNA; ANA: nuclear antibodies.

**Table 2:** Selection of Epstein-Barr virus proteins and their functions [33, 39].

Function	Protein
Latent state nuclear antigens	EBNA1
	EBNA-LP
	EBNA2
	EBNA3A
	EBNA3B
	EBNA3C

Glycoproteins involved in viral entry	gp350
	gp42
	BMRF2
	gH
	gL
	gp110

Envelope proteins	gN
	gp150
	BILF2
	BILF1
	BDLF2

Initiation of lytic replication	BZLF1
	BRLF1
	EA/D
	BSLF1
	BBLF4
	BBLF2/3
	BALF5
	BALF2

Immune evasion	Viral IL10
	BARF1
	LF2
	BNLF2a
	BMLF1/BSLF2

Antiapoptotic	EA/R
	BALF1
	LMP1
	LMP2
